# Early silica crust formation in planetesimals by metastable silica-rich liquid immiscibility or cristobalite crystallisation: the possible origin of silica-rich chondrules

**DOI:** 10.1038/s41598-020-61806-5

**Published:** 2020-03-16

**Authors:** François Faure

**Affiliations:** Université de Lorraine, CNRS, CRPG, UMR 7358, 15 rue Notre Dame des Pauvres, F-54501 Vandoeuvre-lès-Nancy, France

**Keywords:** Early solar system, Meteoritics, Petrology

## Abstract

The formation and differentiation processes of planetesimals—small bodies in the solar system—remain actively debated. Planetesimal differentiation is known to have occurred early (<1.5 Myr after the formation of Ca-Al-rich inclusions), as attested by the ages of iron meteorites. Metal-silicate segregation implies global-scale melting, induced by heat released from short-lived radiogenic isotopes, and the consequent generation of a silicate magma ocean. Thermodynamic calculations show that silicate magma crystallisation would have induced silicate-silicate differentiation, leading to the formation of a thick olivine-dominated “mantle” and a thin basaltic “crust”. However, thermodynamic modelling of magma ocean crystallisation does not produce any silica phases. Here I experimentally show that crystallisation of a chondritic liquid does not follow the thermodynamically predicted path. Silica phases are generated early (before 55% differentiation) as a function of initial magma ocean temperature. As cristobalite or liquid silica phases are less dense than residual liquids or olivine, silica phases could have formed proto-crusts that would have acted as buoyant lids at the surfaces of planetesimals, allowing the eventual accretion and preservation of debris (chondrites). Moreover, the destruction of such a crust by impacts could provide an explanation for the origin of the silica reservoir that condensed around some chondrules.

## Introduction

It is largely accepted that all large differentiated solar system bodies, including Earth, experienced at least one magma ocean stage^1,2^. For smaller bodies such as planetesimals, the existence of a magma ocean requires that the body attained a minimum size and formed sufficiently early (<1–2 Myr after the formation of Ca-Al-rich inclusions, CAIs) to have had enough ^26^Al to supply radiogenic heat for melting^3,4^. Substantial evidence suggests that a large-scale melting phase also occurred in small early differentiated bodies such as Vesta^5^ or the angrite parent body^6^. For instance, the recent Dawn mission confirmed the presence of a metallic core within Vesta^7^. Similarly, the ages of iron meteorites (which formed <1 Myr after CAIs) confirm both the existence of bodies large enough to melt and that melting must have occurred on a large scale^8,9^. However there is no consensus on whether planetesimals formed from the aggregation and differentiation of primitive materials formed previously in the nebular gas (i.e., chondrites, principally composed of chondrules) or chondrules formed during impacts between pre-existing planetesimals (see Connolly and Jones^10^ for a recent review). An intermediate model suggests that the two processes occurred simultaneously^11^, consistent with the fact that planetesimals and chondrules record the same ages of formation. Whatever the model considered, planetesimal differentiation occurred early, as constrained by the ages of iron meteorites^9^.

Thermodynamic calculations of magma ocean crystallisation on small bodies like Vesta are relatively simple because the pressure at the bottom of the magma ocean is estimated to have been inferior to 1 kbar; thus, the adiabatic temperature and solidus change little with depth, i.e., by less than 4 °C and 20 °C, respectively^12^. The crystallisation path during equilibrium or fractional crystallisation can be determined using stable phase diagrams or thermodynamic modelling^13,14^ according to processes well known since Bowen’s^15^ pioneering studies. Differentiation sequences determined for chondritic magmas produce dunite (i.e., only olivine) at the beginning of crystallisation followed by variable amounts of harzbugite (olivine + orthopyroxene), and the end of differentiation is marked by the production of basaltic magma^16^. This crystallisation sequence results because chondrite compositions, excluding enstatite chondrites, show comparable atomic abundances of Mg, Fe, and Si. Thus, even if a large quantity of Fe has already been segregated into the core or is in its reduced state, phase relations at equilibrium imply that only olivine and pyroxene should crystallise with this composition; silica phases are not stable^17^. However, this differentiation sequence has never been tested experimentally for chondritic compositions in a completely molten body, as expected in the case of magma oceans. Here I show, in contrast, that silica phases can be produced early during magma ocean crystallisation because pyroxene fails to nucleate.

## Results and discussions

**Figure 1:**
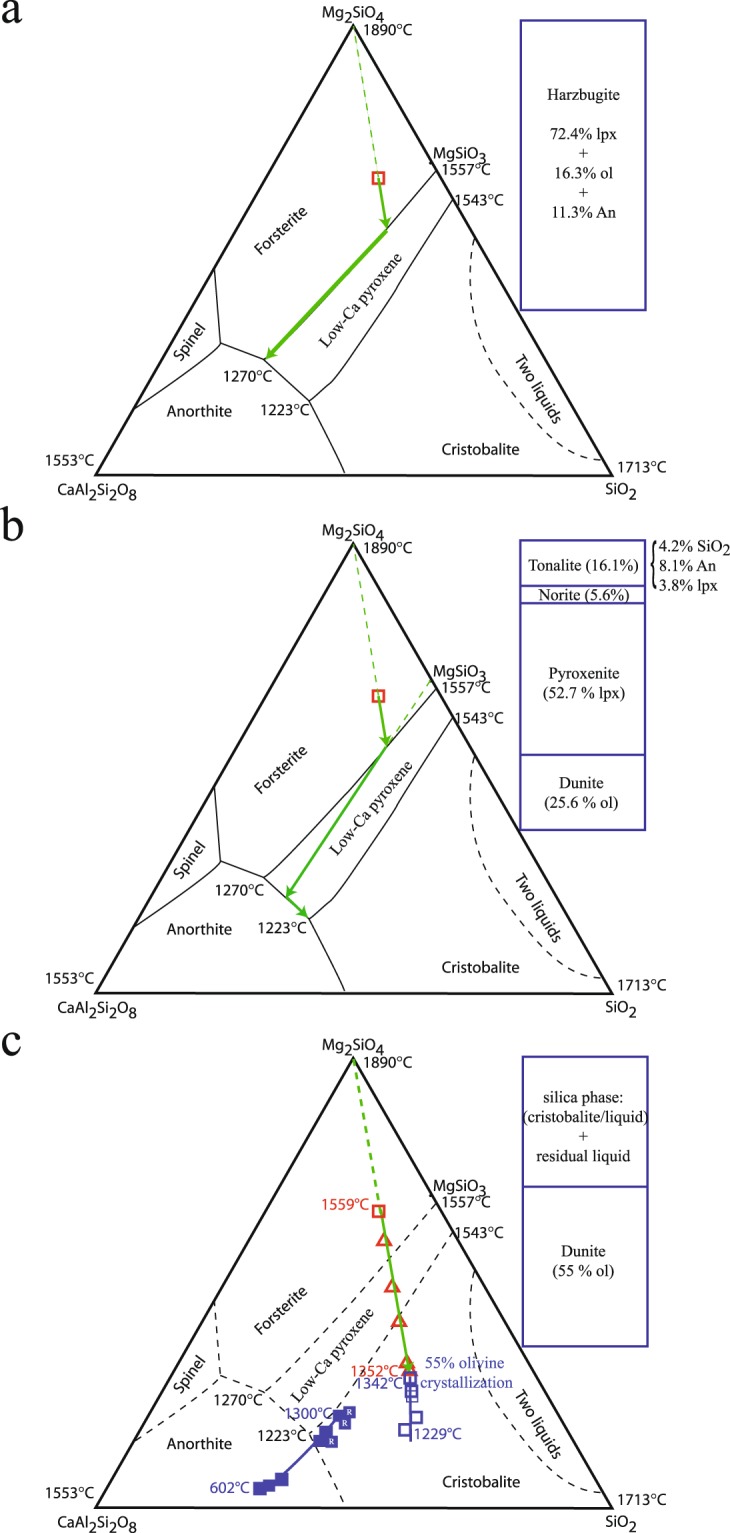
Chemical compositions of the liquid line of descent projected from diopside onto the SiO_2_-forsterite-anorthite pseudoternary liquidus phase diagram during crystallisation of the CI* starting composition (open red square) and the mineral assemblages and associated rock types formed (outset rectangles). Predicted (**a**) melt equilibrium and (**b**) melt fractionation paths (green arrows) and (**c**) experimental liquid lines of descent obtained for metastable crystallisation of CI*. Chemical compositions of residual liquids in stable equilibrium are represented by open red triangles, and those of residual liquids in metastable equilibrium with immiscible silica-rich liquids or cristobalite crystals are represented by open and filled blue squares, respectively. ‘R’ inside filled blue squares indicates reverse experiments. Abbreviations: lpx, low-Ca pyroxene; ol, olivine; and An, anorthite.

I performed slow-cooling experiments (1–2 °C/h to ensure that the crystals and liquid remained under near-equilibrium conditions^18^) on a liquid with a simplified CaO-MgO-Al_2_O_3_-SiO_2_ composition (referred to here as CI*) that mimics the CI chondrite composition (see Methods for justification of this simplified iron-free starting composition). The experimental conditions and results are detailed in Supplementary Tables S1–S4. The stable differentiation path for this composition is presented for both equilibrium (Fig. 1a) and fractional crystallisation (Fig. 1b). Only fractional crystallisation produced a small quantity (<5%) of cristobalite or tridymite crystals at the end of differentiation.

The most important results of these experiments are as follows. First, charges quenched in the pyroxene stability field show exclusively olivine, indicating that the reaction olivine + Liq ⇒ low-Ca pyroxene did not occur. Second, after sufficient cooling, a silica phase appears in addition to olivine and the residual liquid. Silica phases are liquid (quenched to glass) or α cristobalite after quenching. Low-Ca pyroxene is sometimes present in charges where a silica phase appeared, but appears to crystallise after cristobalite. Two different liquid lines of descent can be produced depending on the silica phase that forms (Fig. 1c).

**Figure 2:**
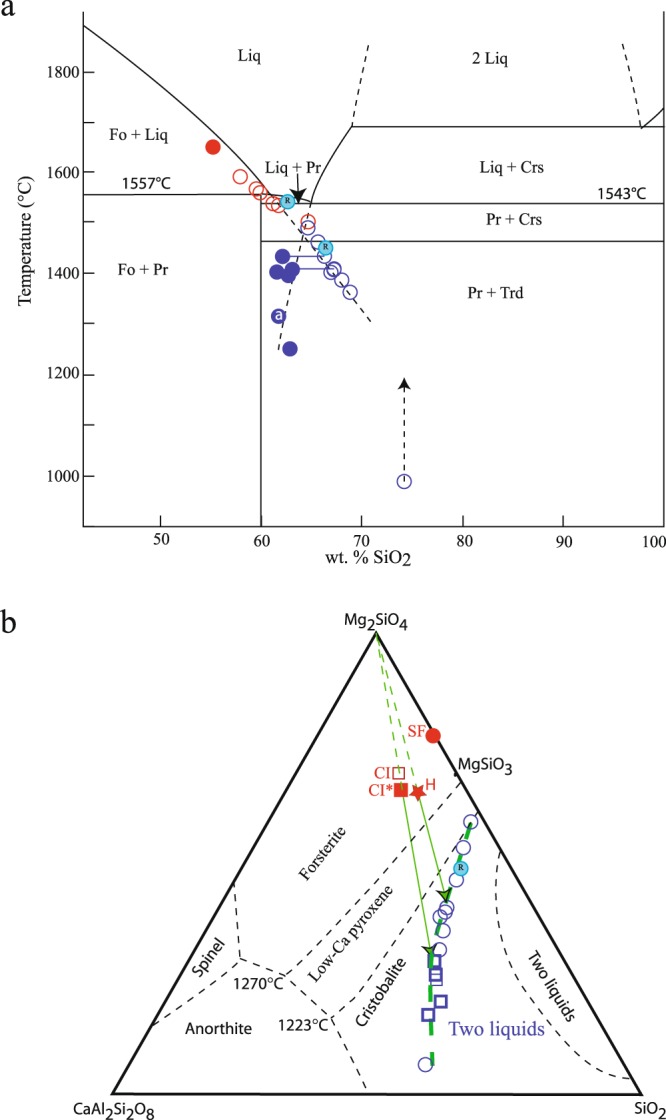
Chemical compositions of the liquid line of descent projected in (**a**) the forsterite-silica binary liquidus phase diagram and (**b**) the SiO_2_-forsterite-anorthite pseudoternary liquidus phase diagram during crystallisation from the SF starting composition. In (**a**), the two metastable liquid lines of descent are obtained depending on which phase is formed. Open and filled blue circles correspond to a metastable liquid in equilibrium with either immiscible silica liquid or cristobalite crystals, respectively. Open red circles show the compositions of stable liquids in equilibrium with forsterite crystals. The SF starting composition is represented by the filled red circle. The position of the open blue circle at 992 °C is probably too low because it should be below the glass transition. It is important to note that the SF composition is not a pure MgO-SiO_2_ mix, but also contains CaO, Al_2_O_3_, and TiO_2_ as minor oxides before crystallisation to increase their concentrations in the residual liquid. The phase diagram for the forsterite-silica portion of the MgO-SiO_2_ system showing the metastable extensions of the forsterite and cristobalite liquidi is modified after Kirkpatrick *et al*.^32^. Abbreviations: Liq, liquid; Crs, cristobalite; Trd, tridymite; Pr, protoenstatite; Fo, forsterite; a, air quench; and R, reverse experiment. In (**b**), the two metastable liquid (immiscibility) field is extended (thick green dashed curved) using data obtained in SF (open blue circles) and CI* (open blue squares) experiments. Only 41% forsterite crystallisation is needed to reach the liquid immiscibility field from an H chondrite starting composition (red star; composition from Newsom^33^). Projections of the chemical compositions of CI chondrite (open red square) and the experimental starting compositions (CI*, filled red square; SF, filled red circle) are shown.

These experiments therefore demonstrate the occurrence of unexpected metastable phase separation mechanisms within the residual liquid; i.e., either silica-rich liquid immiscibility or cristobalite crystallisation. These two different results depend on the activated metastable liquidus curve (Fig. 2a), as demonstrated experimentally with a more simplified composition (SF) near the silica-forsterite binary, but slightly doped with Al, Ca, and Ti as minor elements (Supplementary Tables S4–S7, Supplementary Fig. S1). It is important to note that liquids on these metastable liquidus curves are under equilibrium or near-equilibrium conditions, as attested by isothermal (durations of several hours to days, procedure C in Methods) and reverse experiments (procedure D in Methods) that give the same results as charges quenched immediately after cooling (Figs. 1c, 2). In contrast, experiments with a starting temperature well below the liquidus temperature, but always in the forsterite stability field and with the same slow cooling rate, produce low-Ca pyroxene (Supplementary Fig. S2). Therefore, the initial temperature, and not the slow cooling rate used in the experiments, is responsible for the observed suppression of pyroxene nucleation.

By combining the results from experiments performed on the CI* and SF starting compositions, the metastable two-liquid (immiscibility) field can be determined, which is larger and at lower temperature than the immiscibility field known to occur at stable equilibrium (Fig. 2b). A key finding from these experiments is that an immiscible, nearly pure silica liquid can be produced directly and fairly early during cooling. Only 55% differentiation is needed to obtain a silica-rich immiscible liquid from the CI* composition (Fig. 1c), much earlier than the 84% differentiation required in the stable domain where the fractional crystallisation model is usually calculated (Fig. 1b). Immiscible silica-rich liquid can be produced even earlier (>41% olivine crystallisation) from an H ordinary chondrite composition (Fig. 2b).

**Figure 3:**
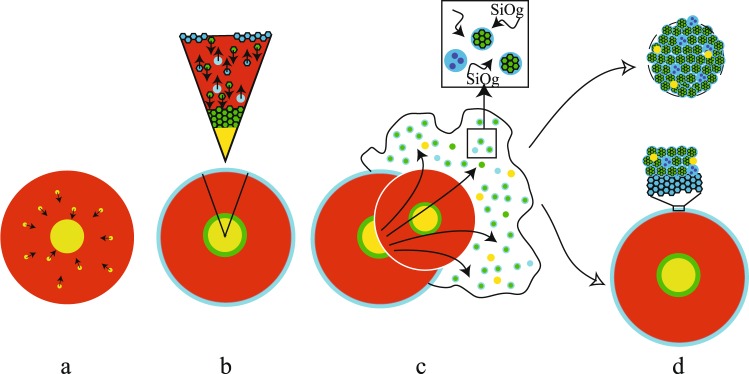
Conceptual sketch of the various planetesimal differentiation steps and chondrule formation by impacting. (**a**) Metal-silicate differentiation during large-scale melting to produce a magma ocean (metal, yellow; silicate, red). (**b**) Crystallisation of the magma ocean initially produces only olivine crystals (dunite cumulate, green) and then forms immiscible silica liquid (blue circles) or cristobalite crystals (blue hexagons), which, due to their lower density compared to olivine, should form a silica crust. (**c**) Collisions between planetesimals produce a silica-rich gas plume that interacts with partially or completely molten silicate droplets to form POP or PP chondrules. Silica-rich chondrules correspond to a sampling of the magma ocean during its immiscible metastable differentiation. (**d**) Previously formed chondrules can either aggregate to form a small parent body or accreted as debris on the silica crust of another planetesimal, from which future chondrites are derived.

Moreover, the large difference between the densities of olivine (3.22 g/cm^3^ for fortserite) and cristobalite (2.32–2.36 g/cm^3^) may induce gravitational segregation. This result is particularly important because the silica phase could buoyantly form a primitive crust around planetesimals, insulating the magma ocean from its cold atmosphere and consequently decreasing its cooling rate (Fig. 3a,b). Moreover, the low density of such a silica crust precludes its foundering into the magma ocean (as expected in the case of a denser basaltic crust^2^), allowing it to accumulate and protect debris from which future chondrites could have derived, as proposed in the model of Elkins-Tanton *et al*.^12^.

Although the presence of silica phases in clasts or chondrules has previously been attributed to a metastable crystallisation process^19,20^, the metastable model has been challenged as it has not been supported by experimental results^21^. The new results presented here show that metastable silica crystallisation during planetesimal differentiation is a viable alternative to fractional condensation from the cooling nebula^22^.

In cases where chondrules result from planetesimal collisions, the existence of metastable silica could therefore explain both the origin of silica-rich chondrules that sometimes evidence silica-silicate immiscibility^23^ and the porphyritic olivine and pyroxene (POP) texture believed to result from the late interaction of chondrules with a silica-rich gas^24^ (Fig. 3c). Regarding the former, silica-rich chondrules would represent the sampling of the magma ocean during its immiscible metastable differentiation at relatively low temperature (<1,500 °C) rather than the result of a very high temperature event (>1,695 °C)^23^ that would have melted virtually every chondrule already formed (1,400 °C < chondrule liquidi <1,750 °C)^25^. Regarding the POP texture, volatilisation of planetesimals’ silica crusts upon collision would have produced a silica gas plume that could have condensed late on relic fragments of cumulate olivine, as described in the model of Libourel and Krot^24^.

Importantly, the occurrence of silica phases is a function of the maximum melting temperature of the planetesimal, which in turn depends on its size and ^26^Al content. However, not all bodies experienced intensive melting, as demonstrated by the acapulcoite and lodranite meteorite groups^26,27^. Experiments with starting temperatures at least 20 °C below the liquidus of the CI* composition produced olivine and pyroxene along liquid lines of descent that are more consistent with the stable thermodynamic diagram; i.e., a peritectic reaction occurred and no silica phase was produced (Supplementary Fig. S2). Therefore, in the case of low degrees of melting, crystallisation of the generated magmas would produce only an olivine-dominated “mantle” and basaltic “crust” according to thermodynamic calculations. However, the most important parameter controlling the delay of pyroxene nucleation is the degree of superheating with respect to this second phase^18^. In these experiments, the degree of superheating relative to the pyroxene liquidus was around 50 °C for both the CI* and SF compositions. Total melting is therefore not required to produce metastable processes.

Silicate liquid immiscibility might not have been restricted to planetesimal differentiation and could also have occurred on the early Earth. Felsic spheroidal structures have been observed in several komatiites—high-Mg, and thus high temperature (1,520–1,560 °C)^28^, lavas that are well documented in Archean (>2.5 Ga) terrains on Earth. Although the origin of these felsic globules is debated, the quenching of immiscible liquids has been proposed^29^. The experimental results shown here demonstrate that silicate liquid immiscibility is a natural consequence of delayed pyroxene nucleation. Indeed, suppressed pyroxene nucleation has been proposed to explain the absence of pyroxene in komatiitic lavas with olivine at the liquidus^30^. Thus, immiscibility could occur in both extraterrestrial and terrestrial magmas that reach very high initial temperatures, i.e. >1,540 °C for the simple CI* composition, or lower temperatures in more complex systems where Fe^2+^ prevents pyroxene crystallisation at low pressure^31^.

## Methods

Two starting compositions were prepared from mixtures of reagent grade oxides, melted above their liquidus temperatures for 6 h in a platinum crucible, and then quenched in water. The CI* composition is a CaO-MgO-Al_2_O_3_-SiO_2_ (CMAS) composition that mimics a CI chondrite composition (Supplementary Table S2). The SF composition corresponds to a more simple composition in the binary forsterite-silica system and includes Al, Ca, and Ti as trace elements (Supplementary Table S6).

The iron-free CI* starting composition was chosen to mimic the CI chondrite composition based on two motivations. First, the aim of these experiments is to demonstrate that silica phases can directly crystallise from an ultrabasic composition despite the fact that stable equilibrium phase relations state that this is not allowed because of the peritectic reaction forsterite (Mg-rich olivine) + liquid ⇒ low-Ca pyroxene. In contrast, iron (FeO) in the starting liquid composition will subdue the peritectic reaction until it disappears completely at sufficient iron content, co-crystallising olivine and a silica phase (Supplementary Fig. S3). Therefore, the CI* CMAS composition was chosen as the worst possible composition to coproduce olivine and silica. Moreover, olivines in type I chondrules, generally considered to be primitive, are rich in magnesium and very poor in iron.

The second motivation to use the simplified CI* CMAS composition was to avoid experimental artefacts that influence the results, such as iron loss into the platinum wire or issues related to controlling oxygen fugacity.

The starting glasses were ground, and aliquots of the ground glasses were pelletized. Pellets were placed on a platinum wire loop (3 mm in diameter) at the end of a ceramic rod and inserted into the hot zone (3 cm of constant temperature) of a 1-atm vertical furnace. The tip of a Pt/PtRh_10_ thermocouple, calibrated against the melting points of gold and palladium, was positioned up to 1 cm above the pellets; this setup is believed to be accurate to ±5 °C.

Four thermal protocols (A–D, below) were used in this study. All experiments were quenched in water, except one experiment that was quenched in air (see Fig. 2).

(A) In isothermal experiments, the experimental charges were held at a predetermined temperature to determine liquidus temperatures or the temperatures at which the second phase (pyroxene) appears.
(B) Dynamic crystallisation experiments were initially held above (protocol B1, corresponding to the majority of dynamic crystallisation experiments) or below (B2) the liquidus temperature for 1 h, then cooled at 1 or 2 °C/h before either quenching or continuation to protocol C.
(C) Some experiments from protocol B were held at the final temperature for several hours or days before quenching to test the stability and equilibrium state of metastable liquids or cristobalite crystals.
(D) Reverse experiments consisted of reheating charges just after procedure B to test metastable equilibrium conditions. Charges were reheated into either the metastable (most reverse experiments) or stable field (one experiment) and held isothermally for several days before quenching.

The results obtained from procedures C and D demonstrate that, whatever the thermal procedure used, the charges were near stable or metastable equilibrium conditions. Therefore, kinetic effects due to cooling are not relevant to explain the observations.

After final quenching, charges were mounted in epoxy and prepared as polished sections. Samples were examined using a scanning electron microscope (JEOL 6510) at the CRPG. Chemical analyses were performed either by wavelength dispersive spectroscopy (WDS) on the Cameca SX100 electron microprobe at the Université de Lorraine (Service Commun de Microscopies Electroniques et de Microanalyses) or by energy dispersive spectroscopy (EDS) on the CRPG JEOL 6510 equipped with a Bruker AXS Xflash detector “Quantax”. The accelerating voltage was 15 kV (both WDS and EDS) and the beam current was either 10 nA (WDS) or 0.25 nA (EDS). Microprobe WDS analyses were calibrated using the following standards: synthetic forsterite for Mg and Si, corundum for Al, and wollastonite for Ca. Copper was used for EDS spectrometer calibration. A relative error of <10% was determined for the semi-quantitative EDS analyses. The silica mineral was first microscopically identified as cristobalite by identification of the characteristic “roof tile” texture and then confirmed by X-ray powder diffraction (Bruker D8 diffractometer with Co Kα radiation).

## Supplementary information

Supplementary information
