# Supplementary material for: Early silica crust formation in planetesimals by metastable silica-rich liquid immiscibility or cristobalite crystallisation: the possible origin of silica-rich chondrules
Source: Sci Rep. 2020 Mar 16;10:4765. doi: 10.1038/s41598-020-61806-5 (PMC7075931; doi:10.1038/s41598-020-61806-5)
Supplement: Supplementary file 1 — Supplementary information [file 41598_2020_61806_MOESM1_ESM.pdf]

Supplementary information

# Early silica crust formation in planetesimals by metastable silica-rich liquid immiscibility or cristobalite crystallisation: the possible origin of silica-rich chondrules.

François Faure

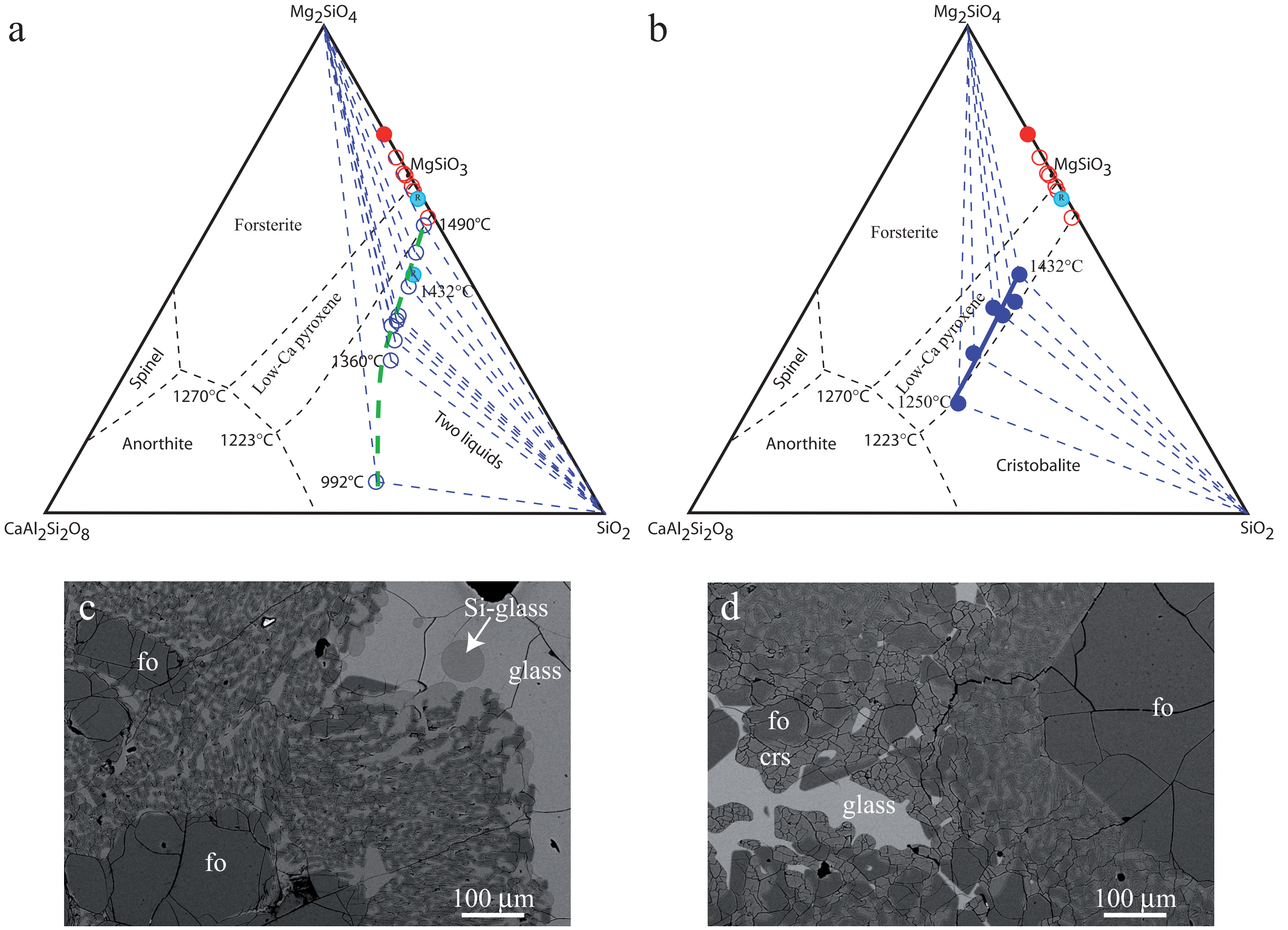


Supplementary Fig. S1: Chemical compositions of the residual liquid line of descent as a function of the silica phase formed, either (a) immiscible silica-rich liquid or (b) cristobalite crystals, projected onto the SiO2-forsterite-anorthite pseudoternary liquidus phase diagram during crystallisation from the SF composition. SEM images showing, for the same experimental conditions (quench at 1,432 °C after being held at the quench temperature for 16.5 h), charges that formed (c) silica-rich immiscible droplets and (d) cristobalite crystals. The SF starting composition is represented by the filled red circle, stable residual liquids by open red circles, metastable residual liquids near equilibrium with immiscible silica liquid by open blue circles, metastable residual liquids near equilibrium with cristobalite crystals by filled blue circles, and the composition of residual liquids after reverse experiments by filled light blue circles labelled 'R'. Abbreviations: fo, forsterite; and crs, cristobalite.

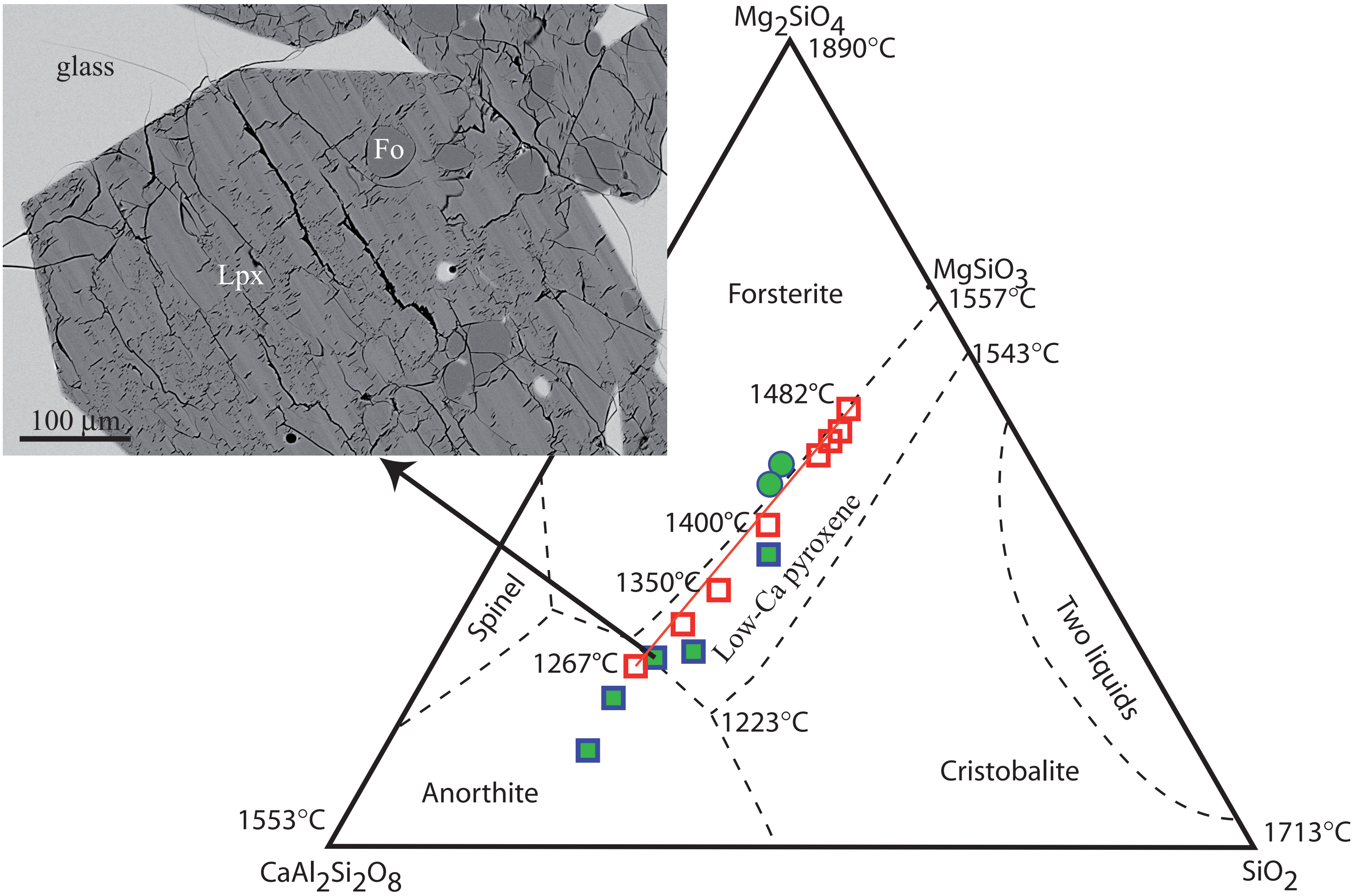


Supplementary Fig. S2: Chemical compositions of residual liquids projected from diopside onto the SiO2-forsterite-anorthite pseudoternary liquidus phase diagram during crystallisation from CI* (blue squares filled green) or SF (blue circles filled green) starting compositions when cooled from an initial temperature below their liquidus temperatures. In this case, large low-Ca pyroxene crystals formed with a poikilitic texture enclosing small olivine crystals (SEM photo of charge CI*-27). Open red squares correspond to the chemical compositions of liquids obtained during isothermal experiments and therefore delimit the peritectic curve for CI*, which is not completely in the plane SiO2-forsterite-anorthite (it is slightly diopside normative).

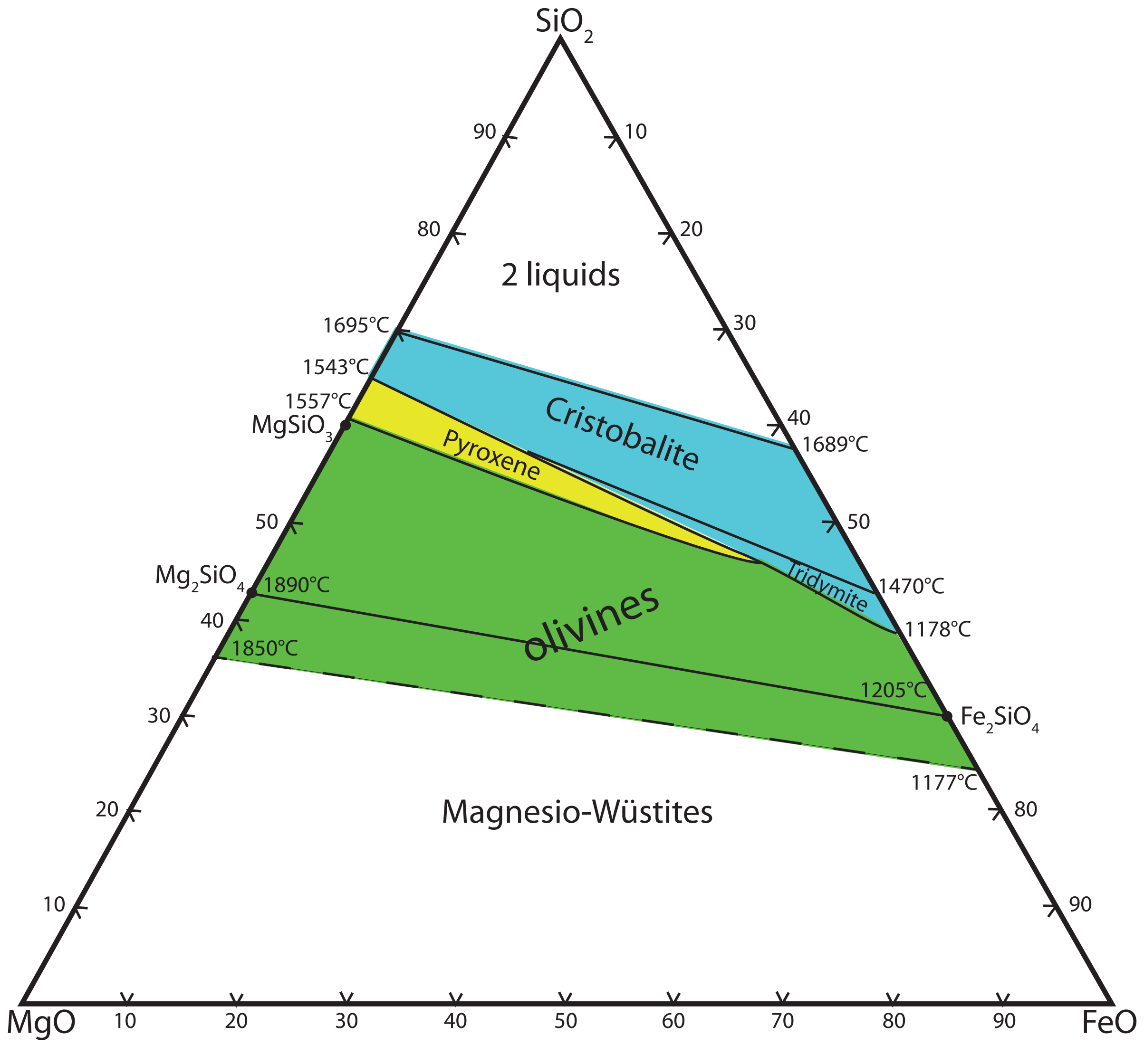


Supplementary Fig. S3: SiO2-MgO-FeO liquidus phase diagram showing the stability field of pyroxene, which separates the fields of olivine from those of silica phases in sufficiently Fe-poor compositions (modified after Bowen and Schairer[31]).

Table S1: Summary of run conditions for isothermal and dynamic crystallisation experiments and products for the CI* starting composition.

| Run n° | $T_{starting}$ (°C) | d$T$/d$t$ (°C/h) | Dwell time before quench (h) | $T_{quench}$ (°C) | Run products |
|---|---|---|---|---|---|
| Isothermal crystallisation experiments | | | | | |
| CI*-1 | 1,579 | 0 | 4.5 | 1,579 | gl |
| CI*-2 | 1,559 | 0 | 14 | 1,559 | gl |
| CI*-3 | 1,558 | 0 | 4.5 | 1,558 | fo, gl |
| CI*-4 | 1,557 | 0 | 19.5 | 1,557 | fo, gl |
| CI*-5 | 1,549 | 0 | 2 | 1,549 | fo, gl |
| CI*-6 | 1,501 | 0 | 6 | 1,501 | fo, gl |
| CI*-7 | 1,491 | 0 | 5 | 1,491 | fo, gl |
| CI*-8 | 1,486 | 0 | 8 | 1,486 | fo, gl |
| CI*-9 | 1,483 | 0 | 90 | 1,483 | fo, gl |
| CI*-10 | 1,482 | 0 | 18 | 1,482 | fo, lpx, gl |
| CI*-11 | 1,461 | 0 | 4 | 1,461 | fo, lpx, gl |
| CI*-12 | 1,451 | 0 | 4 | 1,451 | fo, lpx, gl |
| CI*-13 | 1,400 | 0 | 15 | 1,400 | fo, lpx, gl |
| CI*-14 | 1,349 | 0 | 15 | 1,349 | fo, lpx, gl |
| CI*-15 | 1,316 | 0 | 24 | 1,316 | fo, lpx, gl |
| CI*-16 | 1,267 | 0 | 96 | 1,267 | fo, lpx, gl |
| Dynamic crystallisation experiments | | | | | |
| CI*-17 | 1,589 | 2 | 0 | 602 | fo, lpx, gl, crs |
| CI*-18 | 1,588 | 2 | 52 | 1,361 | fo, gl |
| CI*-19 | 1,588 | 2 | 41 | 1,352 | fo, gl |
| CI*-20 | 1,588 | 2 | 25 | 1,342 | fo, 2 gl |
| CI*-21 | 1,586 | 2 | 14 | 1,322 | fo, 2 gl |
| CI*-22 | 1,586 | 2 | 0 | 1,086 | fo, lpx, gl, crs |
| CI*-23 | 1,585 | 2 | 0 | 1,509 | fo, gl |
| CI*-24 | 1,585 | 2 | 20 | 1,455 | fo, gl |
| CI*-25 | 1,585 | 2 | 0 | 1,403 | fo, gl |
| CI*-26 | 1,585 | 2 | 0 | 1,302 | fo, 2 gl |
| CI*-27-A | 1,585 | 2 | 264 | 1,300 | fo, 2 gl |
| CI*-27-B | 1,585 | 2 | 264 | 1,300 | fo, lpx, gl, crs |
| CI*-28 | 1,585 | 2 | 0 | 1,264 | fo, lpx, gl, crs |
| CI*-29 | 1,585 | 2 | 0 | 1,248 | fo, lpx, gl, crs |
| CI*-30 | 1,569 | 2 | 0 | 959 | fo, lpx, gl, crs |
| CI*-31 | 1,550 | 2 | 0 | 1,417 | fo, gl |
| CI*-32 | 1,549 | 2 | 0 | 1,229 | fo, 2 gl |
| CI*-33 | 1,530 | 2 | 17.5 | 1,280 | fo, lpx, gl |
| CI*-34 | 1,525 | 2 | 47 | 1,390 | fo, lpx, gl |
| CI*-35 | 1,505 | 2 | 32 | 1,296 | fo, lpx, gl |
| CI*-36 | 1,505 | 2 | 37 | 1,100 | fo, lpx, gl |
| CI*-37 | 1,500 | 2 | 157 | 1,246 | fo, lpx, gl |

Abbreviations: gl, glass; fo, forsterite; lpx, low-Ca pyroxene; crs, cristobalite. The suffix A or B indicates that two charges from the same experiment displayed different results.

Table S2: Chemical compositions (in wt. %) and normative mineralogy of the bulk CI* starting composition (experiment CI*-1) and the various residual liquids (glass) obtained during crystallisation of CI*.

| Run n° | *n* | $SiO_2$ | $Al_20_3$ | MgO | CaO | An | Di | Fo | Qtz |
|---|---|---|---|---|---|---|---|---|---|
| CI*-1 | 6 | 55.16 (0.12) | 4.28 (0.06) | 37.24 (0.07) | 3.31 (0.02) | 11.69 | 3.68 | 63.80 | 20.82 |
| CI*-5 | 5 | 56.79 (0.07) | 4.84 (0.06) | 35.03 (0.07) | 3.33 (0.03) | 13.21 | 2.57 | 60.30 | 23.90 |
| CI*-6 | 6 | 59.31 (0.08) | 5.54 (0.06) | 31.20 (0.07) | 3.93 (0.04) | 15.14 | 3.39 | 53.35 | 28.10 |
| CI*-7 | 5 | 59.43 (0.17) | 5.58 (0.06) | 30.96 (0.14) | 4.00 (0.03) | 15.24 | 3.60 | 52.87 | 28.27 |
| CI*-8 | 5 | 60.85 (0.17) | 5.75 (0.07) | 29.05 (0.14) | 4.33 (0.04) | 15.69 | 4.51 | 49.24 | 30.54 |
| CI*-9[a] | 5 | 59.30 (0.16) | 5.87 (0.08) | 30.73 (0.06) | 4.08 (0.03) | 16.02 | 3.30 | 52.56 | 28.10 |
| CI*-10[a] | 5 | 59.42 (0.19) | 5.89 (0.11) | 30.52 (0.05) | 4.15 (0.10) | 16.07 | 3.55 | 52.12 | 28.24 |
| CI*-11[a] | 4 | 59.32 (0.13) | 7.27 (0.21) | 28.18 (0.39) | 5.21 (0.15) | 19.83 | 4.70 | 47.66 | 27.79 |
| CI*-12[a] | 7 | 59.11 (0.17) | 7.93 (0.09) | 27.20 (0.11) | 5.75 (0.10) | 21.65 | 5.35 | 45.73 | 27.25 |
| CI*-13[a] | 9 | 58.50 (0.16) | 10.98 (0.05) | 22.31 (0.28) | 8.19 (0.12) | 29.97 | 8.31 | 36.24 | 25.46 |
| CI*-14[a] | 5 | 57.67 (0.08) | 13.77 (0.05) | 18.31 (0.04) | 10.24 (0.07) | 37.58 | 10.28 | 28.61 | 23.50 |
| CI*-15[a] | 5 | 56.84 (0.09) | 15.41 (0.15) | 16.08 (0.18) | 11.64 (0.17) | 42.06 | 12.24 | 24.10 | 21.58 |
| CI*-16[a] | 5 | 55.64 (0.11) | 17.50 (0.09) | 13.47 (0.06) | 13.37 (0.08) | 47.76 | 14.47 | 18.80 | 18.95 |
| CI*-17 | 4 | 59.85 (0.56) | 22.91 (0.50) | 3.24 (0.13) | 13.98 (0.07) | 62.51 | 5.36 | 3.91 | 28.20 |
| CI*-18 | 10 | 67.13 (0.60) | 7.98 (0.32) | 18.68 (0.17) | 6.19 (0.19) | 21.77 | 6.98 | 30.33 | 40.90 |
| CI*-19 | 11 | 67.98 (0.25) | 8.09 (0.07) | 17.58 (0.25) | 6.34 (0.06) | 22.08 | 7.29 | 28.31 | 42.30 |
| CI*-20 | 11 | 68.30 (0.18) | 8.29 (0.14) | 16.81 (0.17) | 6.59 (0.07) | 22.62 | 7.84 | 26.79 | 42.73 |
| CI*-21 | 5 | 69.21 (0.14) | 8.68 (0.07) | 15.21 (0.05) | 6.88 (0.14) | 23.69 | 8.14 | 23.91 | 44.24 |
| CI*-22 | 6 | 61.10 (0.73) | 19.42 (0.37) | 5.53 (0.30) | 13.93 (0.38) | 53.00 | 12.54 | 5.58 | 28.86 |
| CI*-23 | 4 | 57.62 (0.10) | 5.00 (0.08) | 33.54 (0.10) | 3.82 (0.09) | 13.64 | 4.15 | 57.19 | 25.00 |
| CI*-24 | 6 | 61.25 (0.20) | 6.21 (0.28) | 27.87 (0.22) | 4.65 (0.11) | 16.95 | 4.79 | 47.08 | 31.15 |
| CI*-25 | 35 | 63.98 (0.22) | 7.01 (0.08) | 23.64 (0.18) | 5.35 (0.07) | 19.14 | 5.78 | 39.38 | 35.68 |
| CI*-26 | 7 | 69.68 | 8.91 | 14.63 | 6.76 | 24.33 | 7.17 | 23.21 | 45.27 |

| | | | | | | | | | |
|---|---|---|---|---|---|---|---|---|---|
| | | (0.67) | (0.22) | (0.79) | (0.14) | | | | |
| CI*-27A | 5 | 71.31 | 9.42 | 12.02 | 7.23 | 25.71 | 7.92 | 18.40 | 47.95 |
| | | (0.14) | (0.06) | (0.06) | (0.09) | | | | |
| CI*-27B | 5 | 62.91 | 13.69 | 12.67 | 10.71 | 37.36 | 12.31 | 18.11 | 32.21 |
| | | (0.25) | (0.13) | (0.16) | (0.13) | | | | |
| CI*-28 | 5 | 62.61 | 14.97 | 10.76 | 11.64 | 40.84 | 13.17 | 14.50 | 31.46 |
| | | (0.23) | (0.10) | (0.03) | (0.22) | | | | |
| CI*-29 | 4 | 62.33 | 15.49 | 10.01 | 12.15 | 42.26 | 14.05 | 12.90 | 30.77 |
| | | (0.25) | (0.12) | (0.09) | (0.12) | | | | |
| CI*-30 | 4 | 60.50 | 21.87 | 3.85 | 13.76 | 59.69 | 6.69 | 4.54 | 29.06 |
| | | (1.06) | (0.75) | (0.18) | (0.39) | | | | |
| CI*-31[a] | 3 | 64.26 | 7.11 | 23.39 | 5.22 | 19.41 | 5.07 | 39.17 | 36.33 |
| | | (0.03) | (0.09) | (0.07) | (0.05) | | | | |
| CI*-32 | 5 | 70.35 | 10.19 | 10.88 | 8.56 | 27.81 | 11.40 | 15.28 | 45.48 |
| | | (0.40) | (0.10) | (0.24) | (0.18) | | | | |
| CI*-33[a] | 4 | 56.43 | 17.28 | 13.93 | 12.34 | 47.15 | 10.98 | 20.75 | 21.10 |
| | | (0.22) | (0.15) | (0.12) | (0.03) | | | | |
| CI*-34[a] | 5 | 59.52 | 11.62 | 20.45 | 8.39 | 31.73 | 7.70 | 33.20 | 27.36 |
| | | (0.06) | (0.09) | (0.06) | (0.09) | | | | |
| CI*-35[a] | 5 | 58.27 | 15.96 | 14.42 | 11.33 | 43.55 | 9.87 | 21.97 | 24.60 |
| | | (0.20) | (0.05) | (0.08) | (0.14) | | | | |
| CI*-36[a] | 6 | 55.77 | 23.69 | 6.87 | 13.65 | 64.65 | 2.41 | 11.21 | 21.71 |
| | | (0.65) | (0.45) | (0.24) | (0.11) | | | | |
| CI*-37[a] | 3 | 55.36 | 18.54 | 11.88 | 14.20 | 50.59 | 15.47 | 15.71 | 18.21 |
| | | (0.12) | (0.16) | (0.02) | (0.08) | | | | |
| CI*-38[a] | 6 | 64.07 | 13.34 | 12.64 | 9.93 | 36.41 | 10.00 | 18.82 | 34.74 |
| | | (0.12) | (0.06) | (0.09) | (0.13) | | | | |
| CI*-39[a] | 5 | 64.28 | 14.09 | 11.42 | 10.19 | 38.44 | 9.45 | 16.86 | 35.22 |
| | | (0.07) | (0.03) | (0.08) | (0.06) | | | | |
| CI*-40[a] | 5 | 63.84 | 15.24 | 9.60 | 11.30 | 41.58 | 11.26 | 13.10 | 34.03 |
| | | (0.08) | (0.05) | (0.09) | (0.06) | | | | |

Abbreviations: *n*, number of analyses; An, anorthite; Di, diopside; Fo, forsterite; Qtz, quartz.

[a] EDS analyses.

Table S3: Chemical compositions (in wt. %) of minerals (olivine, fo; low-Ca pyroxene, lpx; cristobalite, crs) or silica-rich liquids (Si-gl) formed during cooling of the CI* starting composition.

| Run n° | phase | *n* | $SiO_2$ | $Al_20_3$ | MgO | CaO |
|---|---|---|---|---|---|---|
| CI*-5 | fo[a] | 5 | 42.97 (0.09) | 0.23 (0.15) | 56.73 (0.10) | 0.05 (0.01) |
| CI*-6 | fo[a] | 5 | 43.33 (0.07) | 0.20 (0.04) | 56.38 (0.10) | 0.07 (0.01) |
| CI*-7 | fo[a] | 5 | 42.98 (0.14) | 0.15 (0.09) | 56.79 (0.16) | 0.06 (0.01) |
| CI*-8 | fo[a] | 5 | 44.83 (0.43) | 0.24 (0.05) | 54.85 (0.42) | 0.06 (0.01) |
| CI*-9 | fo[a] | 5 | 42.61 (0.17) | 0.25 (0.09) | 57.05 (0.14) | 0.07 (0.02) |
| CI*-10 | fo[a] | 5 | 42.60 (0.10) | 0.24 (0.11) | 57.09 (0.09) | 0.05 (0.02) |
| CI*-10 | lpx[a] | 5 | 59.17 (0.17) | 0.57 (0.15) | 40.13 (0.08) | 0.11 (0.01) |
| CI*-11 | fo[a] | 5 | 42.82 (0.17) | 0.10 (0.19) | 56.95 (0.09) | 0.11 (0.02) |
| CI*-11 | lpx[a] | 5 | 59.57 (0.08) | 0.28 (0.07) | 39.91 (0.09) | 0.22 (0.08) |
| CI*-12 | fo[a] | 5 | 42.85 (0.15) | 0.18 (0.12) | 56.86 (0.08) | 0.08 (0.04) |
| CI*-12 | lpx[a] | 6 | 59.44 (0.16) | 0.39 (0.10) | 39.96 (0.11) | 0.19 (0.06) |
| CI*-13 | fo[a] | 6 | 43.49 (0.15) | 0.26 (0.04) | 56.06 (0.21) | 0.17 (0.03) |
| CI*-13 | lpx[a] | 5 | 59.94 (0.56) | 0.50 (0.20) | 39.27 (0.42) | 0.27 (0.02) |
| CI*-14 | fo[a] | 5 | 42.90 (0.07) | 0.15 (0.16) | 56.79 (0.16) | 0.15 (0.03) |
| CI*-14 | lpx[a] | 5 | 59.29 (0.13) | 0.70 (0.21) | 39.54 (0.12) | 0.45 (0.02) |
| CI*-15 | fo[a] | 5 | 43.23 (0.17) | 0.19 (0.18) | 56.35 (0.10) | 0.21 (0.06) |
| CI*-15 | lpx[a] | 4 | 59.54 (0.27) | 1.05 (0.26) | 38.97 (0.33) | 0.43 (0.01) |
| CI*-16 | fo[a] | 3 | 43.14 (0.20) | 0.15 (0.10) | 56.43 (0.24) | 0.26 (0.14) |
| CI*-16 | lpx[a] | 3 | 59.17 (0.10) | 1.18 (0.26) | 39.08 (0.20) | 0.55 (0.03) |
| CI*-17 | fo | 4 | 41.51 (0.11) | 0.012 (0.009) | 58.39 (0.12) | 0.07 (0.02) |
| CI*-17 | lpx | 5 | 58.34 (0.15) | 0.48 (0.19) | 40.67 (0.39) | 0.48 (0.12) |
| CI*-17 | cpx | 3 | 51.43 (0.52) | 6.51 (0.40) | 19.39 (0.31) | 22.65 (0.04) |
| CI*-17 | crs | 4 | 98.86 (0.26) | 0.71 (0.17) | 0.03 (0.03) | 0.37 (0.09) |
| CI*-18 | fo[a] | 5 | 43.34 (0.09) | 0.19 (0.10) | 56.38 (0.10) | 0.06 (0.03) |
| CI*-19 | fo[a] | 5 | 43.93 (0.14) | 0.18 (0.11) | 55.82 (0.15) | 0.05 (0.01) |
| CI*-20 | fo[a] | 5 | 43.26 (0.04) | 0.15 (0.10) | 56.49 (0.11) | 0.08 (0.03) |
| CI*-20 | Si-gl | 4 | 95.39 (0.50) | 2.01 (0.13) | 1.69 (0.35) | 0.89 (0.03) |
| CI*-21 | fo[a] | 5 | 43.21 (0.07) | 0.21 (0.10) | 56.49 (0.07) | 0.07 (0.01) |
| CI*-21 | Si-gl | 3 | 94.20 (0.18) | 2.41 (0.01) | 2.11 (0.18) | 1.25 (0.01) |
| CI*-22 | fo[a] | 5 | 43.60 (0.04) | 0.17 (0.12) | 56.12 (0.13) | 0.10 (0.02) |
| CI*-22 | lpx[a] | 5 | 60.07 (0.08) | 0.53 (0.08) | 38.92 (0.15) | 0.45 (0.03) |
| CI*-22 | cpx[a] | 5 | 53.21 (0.75) | 6.37 (0.79) | 18.53 (1.48) | 21.87 (1.48) |
| CI*-22 | crs | 2 | 98.82 (0.31) | 0.76 (0.23) | 0 | 0.40 (0.07) |
| CI*-23 | fo[a] | 5 | 43.35 (0.09) | 0.09 (0.04) | 56.48 (0.11) | 0.06 (0.01) |
| CI*-24 | fo | 4 | 42.21 (0.22) | 0.01 (0.02) | 57.69 (0.21) | 0.07 (0.01) |
| CI*-25 | fo | 5 | 41.71 (0.16) | 0.05 (0.01) | 58.15 (0.18) | 0.07 (0.02) |
| CI*-26 | fo | 3 | 42.34 (0.47) | 0.17 (0.26) | 57.35 (0.79) | 0.12 (0.11) |
| CI*-26 | Si-gl | 2 | 92.94 (2.56) | 2.34 (0.06) | 3.45 (2.58) | 1.25 (0.04) |
| CI*-27A | fo[a] | 5 | 43.42 (0.12) | 0.17 (0.04) | 56.33 (0.08) | 0.05 (0.01) |
| CI*-27A | Si-gl | 3 | 94.34 (0.02) | 2.55 (0.02) | 1.62 (0.01) | 1.48 (0.03) |
| CI*-27B | fo[a] | 5 | 43.50 (0.12) | 0.18 (0.09) | 56.21 (0.16) | 0.09 (0.04) |
| CI*-27B | lpx[a] | 6 | 60.26 (0.18) | 0.47 (0.18) | 38.94 (0.12) | 0.31 (0.03) |
| CI*-27B | crs | 4 | 99.23 (0.06) | 0.47 (0.05) | 0.02 (0.02) | 0.25 (0.03) |
| CI*-28 | fo[a] | 5 | 43.07 (0.08) | 0.13 (0.08) | 56.74 (0.15) | 0.04 (0.02) |
| CI*-28 | lpx[a] | 7 | 59.61 (0.21) | 0.84 (0.25) | 39.07 (0.25) | 0.46 (0.06) |

| | | | | | | |
|---|---|---|---|---|---|---|
| CI*-28 | crs | 3 | 98.17 (0.12) | 1.12 (0.04) | 0.10 (0.05) | 0.59 (0.07) |
| CI*-29 | fo[a] | 5 | 43.97 (0.11) | 0.16 (0.04) | 55.77 (0.09) | 0.07 (0.01) |
| CI*-29 | lpx[a] | 5 | 60.49 (0.09) | 0.56 (0.17) | 38.50 (0.18) | 0.43 (0.05) |
| CI*-29 | crs | 4 | 98.88 (0.40) | 0.68 (0.22) | 0.09 (0.09) | 0.34 (0.09) |
| CI*-30 | fo[a] | 5 | 44.24 (0.12) | 0.25 (0.06) | 55.37 (0.13) | 0.12 (0.03) |
| CI*-30 | lpx[a] | 5 | 60.45 (0.46) | 0.87 (0.51) | 38.26 (0.40) | 0.41 (0.07) |
| CI*-30 | cpx[a] | 5 | 52.21 (1.07) | 7.37 (1.84) | 17.52 (1.28) | 22.89 (0.58) |
| CI*-31 | fo[a] | 4 | 43.30 (0.16) | 0.16 (0.06) | 56.45 (0.19) | 0.07 (0.02) |
| CI*-32 | fo[a] | 5 | 43.22 (0.09) | 0.16 (0.10) | 56.54 (0.08) | 0.06 (0.02) |
| CI*-32 | Si-gl[a] | 7 | 88.15 (3.39) | 2.66 (0.14) | 7.92 (3.51) | 1.25 (0.08) |
| CI*-33 | fo[a] | 7 | 43.49 (0.22) | 0.13 (0.07) | 56.21 (0.18) | 0.15 (0.02) |
| CI*-33 | lpx[a] | 5 | 60.29 (0.11) | 0.42 (0.13) | 39.11 (0.08) | 0.16 (0.06) |
| CI*-34 | fo[a] | 5 | 43.94 (0.12) | 0.18 (0.05) | 55.75 (0.16) | 0.11 (0.02) |
| CI*-34 | lpx[a] | 5 | 60.72 (0.13) | 0.24 (0.13) | 38.88 (0.16) | 0.15 (0.01) |
| CI*-35 | fo[a] | 5 | 43.93 (0.16) | 0.18 (0.02) | 55.69 (0.14) | 0.18 (0.03) |
| CI*-35 | lpx[a] | 5 | 60.86 (0.25) | 0.35 (0.19) | 38.52 (0.06) | 0.25 (0.10) |
| CI*-36 | fo[a] | 7 | 43.64 (0.46) | 0.21 (0.07) | 55.95 (0.48) | 0.18 (0.04) |
| CI*-36 | lpx[a] | 5 | 59.82 (0.33) | 1.09 (0.29) | 38.62 (0.25) | 0.45 (0.09) |
| CI*-36 | cpx[a] | 10 | 51.34 (0.74) | 9.04 (1.42) | 17.90 (0.58) | 21.69 (0.82) |
| CI*-37 | fo[a] | 7 | 43.23 (0.12) | 0.15 (0.04) | 56.44 (0.14) | 0.17 (0.02) |
| CI*-37 | lpx[a] | 5 | 60.27 (0.12) | 0.22 (0.12) | 39.33 (0.13) | 0.16 (0.05) |
| CI*-38 | fo[a] | 5 | 43.24 (0.17) | 0.22 (0.07) | 56.43 (0.23) | 0.08 (0.02) |
| CI*-38 | lpx[a] | 9 | 59.93 (1.00) | 0.52 (0.09) | 39.15 (0.96) | 0.37 0.06) |
| CI*-38 | crs[a] | 5 | 98.66 (0.10) | 1.00 (0.08) | 0.05 (0.06) | 0.26 (0.06) |
| CI*-39 | fo[a] | 4 | 43.26 (0.10) | 0.27 (0.02) | 56.39 (0.08) | 0.06 (0.02) |
| CI*-39 | lpx[a] | 3 | 59.59 (0.11) | 0.42 (0.06) | 39.56 (0.14) | 0.41 (0.05) |
| CI*-39 | crs[a] | 4 | 98.61 (0.27) | 1.05 (0.20) | 0.07 (0.08) | 0.25 (0.06) |
| CI*-40 | fo[a] | 5 | 43.22 (0.11) | 0.16 (0.11) | 56.53 (0.09) | 0.08 (0.01) |
| CI*-40 | lpx[a] | 5 | 59.67 (0.07) | 0.62 (0.18) | 39.24 (0.17) | 0.45 (0.04) |
| CI*-40 | crs[a] | 5 | 98.09 (0.44) | 1.39 (0.27) | 0.12 (0.07) | 0.38 (0.11) |

Abbreviations: *n*, number of analyses.

[a] EDS analyses.

Table S4: Summary of run conditions for reverse experiments and products obtained with CI* and SF starting compositions.

| Run n° | $T_{starting}$ (°C) | Cooling rate (°C/h) | Dwell temperature (°C) | Reheating rate (°C/h) | Dwell time before quench (h) | $T_{quench}$ (°C) | Run products |
|---|---|---|---|---|---|---|---|
| CI*-38 | 1,586 | 2 | 1,200 | 10 | 171 | 1,311 | fo, lpx, gl, crs |
| CI*-39 | 1,586 | 2 | 1,200 | 10 | 128 | 1,291 | fo, lpx, gl, crs |
| CI*-40 | 1,586 | 2 | 1,200 | 10 | 65 | 1,250 | fo, lpx, gl, crs |
| SF-25 | 1,646 | 2 | 1,400 | 10 | 0 | 1,540 | fo, gl |
| SF-26 | 1,645 | 2 | 1,407 | 10 | 135 | 1,450 | fo, 2 gl |

Abbreviations: gl, glass; fo, forsterite; lpx, low-Ca pyroxene; crs, cristobalite.

Table S5: Summary of run conditions for isothermal and dynamic crystallisation experiments and products for the SF starting composition.

| Run n° | $T_{starting}$ (°C) | d$T$/d$t$ (°C/h) | Dwell time before quench (h) | $T_{quench}$ (°C) | Run products |
|---|---|---|---|---|---|
| Isothermal crystallisation experiments | | | | | |
| SF-1 | 1,648 | 0 | 1 | 1,648 | gl |
| SF-2 | 1,638 | 0 | 1 | 1,638 | fo, gl |
| SF-3 | 1,608 | 0 | 1.5 | 1,608 | fo, gl |
| SF-4 | 1,575 | 0 | 1 | 1,575 | fo, gl |
| Dynamic crystallisation experiments | | | | | |
| SF-5 | 1,648 | 2 | 0 | 1,407 | fo, 2 gl |
| SF-6 | 1,648 | 2 | 0 | 1,385 | fo, 2 gl |
| SF-7-A | 1,647 | 2 | 264 | 1,401 | fo, 2 gl |
| SF-7-B | 1,647 | 2 | 264 | 1,401 | fo, gl, crs |
| SF-8 | 1,646 | 2 | 0 | 1,588 | fo, gl |
| SF-9 | 1,646 | 2 | 0 | 1,558 | fo, gl |
| SF-10 | 1,646 | 2 | 48 | 1,535 | fo, gl |
| SF-11 | 1,646 | 2 | 0 | 1,462 | fo, 2 gl |
| SF-12 | 1,646 | 2 | 0 | 1,360 | fo, 2 gl |
| SF-13 | 1,626 | 2 | 0 | 1,406 | fo, gl, crs |
| SF-14 | 1,626 | 1 | 82 | 1,300 | fo, 2 gl |
| SF-15 | 1,626 | 2 | 0 | 1,312 | fo, gl, crs |
| SF-16-A | 1,626 | 2 | 602 | 1,250 | fo, gl, crs |
| SF-16-B | 1,626 | 2 | 602 | 1,250 | fo, 2 gl, lpx |
| SF-17 | 1,625 | 2 | 18.5 | 1,530 | fo, gl |
| SF-18 | 1,625 | 2 | 87 | 1,499 | fo, gl |
| SF-19 | 1,625 | 2 | 50.5 | 1,490 | fo, 2 gl |
| SF-20-A | 1,625 | 2 | 14.5 | 1,432 | fo, gl, crs |
| SF-20-B | 1,625 | 2 | 14.5 | 1,432 | fo, 2 gl |
| SF-21 | 1,606 | 2 | 0 | 1,566 | fo, gl |
| SF-22 | 1,606 | 2 | 0 | 1,393 | fo, gl, crs |
| SF-23 | 1,597 | 2 | 0 | 1,426 | fo, lpx, gl |
| SF-24 | 1,590 | 2 | 0 | 1,411 | fo, lpx, gl |

Abbreviations: gl, glass; fo, forsterite; lpx, low-Ca pyroxene; crs, cristobalite. The suffix A or B indicates that two charges from the same experiment displayed different results.

Table S6: Chemical compositions (in wt. %) and normative mineralogy of the bulk SF starting composition (experiment SF-1) and the various residual liquids (glass) obtained during crystallisation of SF.

| Run n° | *n* | $SiO_2$ | $TiO_2$ | $Al_2O_3$ | MgO | CaO | An | Di | Fo | Qtz |
|---|---|---|---|---|---|---|---|---|---|---|
| SF-1 | 8 | 55.11 | 0.05 | 0.27 | 44.49 | 0.06 | 0.75 | -0.31 | 77.79 | 21.77 |
| | | (0.34) | (0.03) | (0.02) | (0.34) | (0.01) | | | | |
| SF-2 | 4 | 57.24 | 0.09 | 0.32 | 42.25 | 0.07 | 0.89 | -0.42 | 73.95 | 25.56 |
| | | (0.25) | (0.01) | (0.02) | (0.25) | (0.04) | | | | |
| SF-3 | 4 | 59.20 | 0.09 | 0.33 | 40.25 | 0.10 | 0.92 | -0.29 | 70.41 | 28.96 |
| | | (0.13) | (0.01) | (0.01) | (0.12) | (0.01) | | | | |
| SF-4 | 15 | 60.29 | 0.06 | 0.28 | 39.28 | 0.07 | 0.78 | -0.33 | 68.70 | 30.84 |
| | | (0.16) | (0.01) | (0.02) | (0.17) | (0.02) | | | | |
| SF-5 | 4 | 67.08 | 1.73 | 6.58 | 22.23 | 2.35 | 18.27 | -4.97 | 41.11 | 45.58 |
| | | (0.32) | (0.03) | (0.10) | (0.28) | (0.13) | | | | |
| SF-6 | 5 | 67.95 | 1.97 | 7.54 | 19.82 | 2.71 | 20.99 | -5.66 | 37.13 | 47.53 |
| | | (0.22) | (0.04) | (0.09) | (0.24) | (0.06) | | | | |
| SF-7-A | 4 | 66.82 | 1.74 | 7.08 | 21.67 | 2.66 | 19.68 | -4.83 | 40.07 | 45.08 |
| | | (0.14) | (0.03) | (0.06) | (0.03) | (0.08) | | | | |
| SF-7-B | 5 | 61.40 | 2.06 | 9.60 | 24.03 | 2.89 | 26.74 | -9.41 | 45.89 | 36.77 |
| | | (0.17) | (0.09) | (0.19) | (0.23) | (0.05) | | | | |
| SF-8 | 23 | 57.82 | 0.09 | 0.33 | 41.64 | 0.09 | 0.90 | -0.32 | 72.85 | 26.55 |
| | | (0.56) | (0.02) | (0.03) | (0.58) | (0.02) | | | | |
| SF-9 | 20 | 59.87 | 0.11 | 0.37 | 39.53 | 0.10 | 1.01 | -0.39 | 69.20 | 30.17 |
| | | (0.19) | (0.02) | (0.03) | (0.20) | (0.03) | | | | |
| SF-10 | 6 | 61.07 | 0.12 | 0.39 | 38.27 | 0.13 | 1.07 | -0.33 | 66.99 | 32.26 |
| | | (0.26) | (0.02) | (0.02) | (0.29) | (0.02) | | | | |
| SF-11 | 7 | 65.51 | 0.66 | 2.60 | 30.62 | 0.87 | 7.14 | -2.19 | 54.36 | 40.67 |
| | | (0.19) | (0.04) | (0.13) | (0.33) | (0.03) | | | | |
| SF-12 | 5 | 68.70 | 2.22 | 8.64 | 17.35 | 3.07 | 24.12 | -6.63 | 33.12 | 49.38 |
| | | (0.21) | (0.04) | (0.03) | (0.17) | (0.05) | | | | |
| SF-13 | 5 | 63.02 | 2.11 | 7.55 | 24.51 | 2.79 | 21.05 | -5.35 | 45.44 | 38.85 |
| | | (0.33) | (0.03) | (0.06) | (0.30) | (0.06) | | | | |
| SF-14 | 5 | 67.12 | 1.38 | 6.26 | 22.90 | 2.31 | 17.34 | -4.44 | 41.97 | 45.12 |
| | | (0.17) | (0.05) | (0.03) | (0.12) | (0.06) | | | | |
| SF-15 | 5 | 61.65 | 3.46 | 13.25 | 17.91 | 3.71 | 37.47 | -14.32 | 37.03 | 39.81 |
| | | (0.11) | (0.05) | (0.17) | (0.16) | (0.03) | | | | |
| SF-16-A[a] | 5 | 62.80 | 2.79 | 15.18 | 12.20 | 7.01 | 42.61 | -5.29 | 23.63 | 39.04 |
| | | (0.10) | (0.08) | (0.03) | (0.05) | (0.03) | | | | |
| SF-16-B[a] | 2 | 82.03 | 1.10 | 5.73 | 8.61 | 2.50 | 15.82 | -2.51 | 16.02 | 70.66 |
| | | (0.27) | (0.02) | (0.05) | (0.36) | (0.06) | | | | |
| SF-17 | 5 | 61.63 | 0.11 | 0.38 | 37.74 | 0.11 | 1.05 | -0.35 | 66.06 | 33.24 |
| | | (0.13) | (0.01) | (0.03) | (0.15) | (0.03) | | | | |
| SF-18 | 8 | 64.64 | 0.15 | 0.44 | 34.62 | 0.13 | 1.22 | -0.44 | 60.65 | 38.56 |
| | | (0.13) | (0.03) | (0.03) | (0.10) | (0.02) | | | | |
| SF-19 | 6 | 64.60 | 0.31 | 0.98 | 33.78 | 0.29 | 2.70 | -0.94 | 59.46 | 38.77 |
| | | (0.20) | (0.08) | (0.02) | (0.14) | (0.03) | | | | |
| SF-20-A | 6 | 62.09 | 1.75 | 6.30 | 27.89 | 1.94 | 17.51 | -6.00 | 51.50 | 36.98 |
| | | (0.20) | (0.03) | (0.06) | (0.25) | (0.07) | | | | |
| SF-20-B | 5 | 66.23 | 1.23 | 4.33 | 26.26 | 1.94 | 11.97 | -1.72 | 46.96 | 42.78 |

| | | | | | | | | | | |
|---|---|---|---|---|---|---|---|---|---|---|
| | | (0.13) | (0.05) | (0.06) | (0.22) | (0.04) | | | | |
| SF-21 | 5 | 59.51 | 0.09 | 0.35 | 39.94 | 0.09 | 0.97 | -0.37 | 69.89 | 29.50 |
| | | (0.15) | (0.03) | (0.02) | (0.14) | (0.02) | | | | |
| SF-22 | 5 | 62.58 | 2.59 | 9.21 | 22.90 | 2.70 | 25.80 | -9.37 | 44.08 | 39.48 |
| | | (0.21) | (0.04) | (0.13) | (0.28) | (0.06) | | | | |
| SF-23 | 5 | 55.73 | 3.08 | 10.74 | 27.06 | 3.37 | 30.25 | -10.10 | 52.01 | 27.83 |
| | | (0.29) | (0.12) | (0.25) | (0.45) | (0.12) | | | | |
| SF-24 | 7 | 55.76 | 3.28 | 11.86 | 25.49 | 3.58 | 33.48 | -11.75 | 49.83 | 28.43 |
| | | (0.11) | (0.05) | (0.15) | (0.17) | (0.06) | | | | |
| SF-25 | 13 | 62.52 | 0.10 | 0.44 | 36.78 | 0.13 | 1.20 | -0.40 | 64.40 | 34.78 |
| | | (0.47) | (0.02) | (0.03) | (0.48) | (0.02) | | | | |
| SF-26[a] | 14 | 66.34 | 0.69 | 3.60 | 27.86 | 1.49 | 9.89 | -1.90 | 49.59 | 42.41 |
| | | (0.09) | (0.08) | (0.31) | (0.19) | (0.21) | | | | |

Abbreviations: *n*, number of analyses; An, anorthite; Di, diopside; Fo, forsterite; Qtz, quartz.
[a] EDS analyses.

Table S7: Chemical compositions (in wt. %) of minerals (olivine, fo; low-Ca pyroxene, lpx; cristobalite, crs) or silica-rich liquids (Si-gl) formed during cooling of the SF starting composition.

| Run n° | phase | *n* | $SiO_2$ | $TiO_2$ | $Al_2O_3$ | MgO | CaO |
|---|---|---|---|---|---|---|---|
| SF-3 | fo[a] | 5 | 43.39 (0.12) | n.d. | 0.28 (0.27) | 56.29 (0.22) | 0.02 (0.01) |
| SF-3 | lpx[a] | 5 | 59.95 (0.10) | 0.08 (0.01) | 0.81 (0.04) | 40.07 (0.07) | 0.07 (0.02) |
| SF-4 | fo[a] | 5 | 43.91 (0.09) | n.d. | 0.28 (0.21) | 55.79 (0.14) | 0.01 (0.01) |
| SF-5 | Si-gl | 7 | 98.45 (1.23) | 0.26 (0.21) | 0.60 (0.52) | 0.59 (0.40) | 0.08 (0.08) |
| SF-5 | fo[a] | 5 | 43.43 (0.12) | n.d. | 0.23 (0.07) | 56.32 (0.10) | 0.01 (0.01) |
| SF-6 | Si-gl | 7 | 97.87 (1.72) | 0.34 (0.30) | 0.84 (0.73) | 0.80 (0.57) | 0.13 (0.12) |
| SF-6 | fo[a] | 5 | 43.11 (0.08) | n.d. | 0.24 (0.11) | 56.63 (0.19) | 0.01 (0.01) |
| SF-7-A | Si-gl | 8 | 97.66 (1.76) | 0.40 (0.31) | 0.87 (0.70) | 0.86 (0.59) | 0.18 (0.15) |
| SF-7-A | fo[a] | 5 | 43.31 (0.11) | n.d. | 0.25 (0.03) | 56.40 (0.13) | 0.01 (0.01) |
| SF-7-B | crs | 6 | 99.46 (0.36) | 0.23 (0.15) | 0.17 (0.14) | 0.07 (0.08) | 0.04 (0.03) |
| SF-7-B | fo[a] | 5 | 43.06 (0.12) | n.d. | 0.17 (0.08) | 56.72 (0.14) | 0.03 (0.02) |
| SF-8 | fo[a] | 5 | 43.50 (0.10) | n.d. | 0.19 (0.05) | 56.29 (0.14) | 0.01 (0.01) |
| SF-9 | fo | 5 | 41.41 (0.10) | n.d. | 0.01 (0.02) | 58.56 (0.09) | 0.01 (0.01) |
| SF-10 | fo | 3 | 41.59 (0.20) | n.d. | 0.01 (0.01) | 58.42 (0.20) | 0 |
| SF-11 | Si-gl | 9 | 99.03 (0.65) | 0.09 (0.07) | 0.24 (0.12) | 0.64 (0.60) | 0.02 (0.02) |
| SF-11 | fo[a] | 5 | 43.29 (0.09) | n.d. | 0.16 (0.10) | 56.51 (0.08) | 0.01 (0.02) |
| SF-12 | Si-gl | 7 | 97.91 (1.89) | 0.38 (0.35) | 0.83 (0.81) | 0.69 (0.56) | 0.16 (0.17) |
| SF-12 | fo[a] | 5 | 43.45 (0.12) | n.d. | 0.23 (0.08) | 56.30 (0.13) | 0.01 (0.01) |
| SF-13 | crs | 8 | 99.68 (0.13) | 0.09 (0.07) | 0.17 (0.05) | 0.03 (0.03) | 0.02 (0.02) |
| SF-13 | fo[a] | 6 | 43.30 (0.12) | n.d. | 0.21 (0.13) | 56.45 (0.22) | 0.01 (0.01) |
| SF-14 | Si-gl | 5 | 97.17 (0.68) | 0.41 (0.09) | 1.14 (0.24) | 1.05 (0.25) | 0.20 (0.08) |
| SF-14 | fo[a] | 5 | 43.89 (0.11) | n.d. | 0.27 (0.10) | 55.82 (0.20) | 0.01 (0.01) |
| SF-15 | crs | 3 | 99.70 (0.05) | 0.12 (0.01) | 0.13 (0.01) | 0.02 (0.02) | 0.02 (0.01) |
| SF-15 | fo[a] | 5 | 44.05 (0.10) | n.d. | 0.35 (0.02) | 55.54 (0.09) | 0.04 (0.01) |
| SF-16-A | crs[a] | 4 | 97.85 (0.64) | 0.41 (0.05) | 1.35 (0.42) | 0.12 (0.01) | 0.25 (0.18) |
| SF-16-A | fo[a] | 5 | 43.76 (0.07) | n.d. | 0.31 (0.26) | 55.88 (0.24) | 0.03 (0.01) |
| SF-16-B | Si-gl[a] | 5 | 97.19 (0.08) | 0.38 (0.04) | 1.82 (0.02) | 0.11 (0.03) | 0.48 (0.02) |
| SF-16-B | fo[a] | 5 | 43.17 (0.02) | n.d. | 0.45 (0.26) | 56.33 (0.25) | 0.02 (0.01) |
| SF-16-B | lpx[a] | 5 | 59.78 (0.09) | n.d. | 1.05 (0.12) | 38.93 (0.05) | 0.22 (0.01) |
| SF-17 | fo[a] | 5 | 43.34 (0.06) | n.d. | 0.13 (0.21) | 56.49 (0.20) | 0.02 (0.01) |
| SF-18 | fo[a] | 5 | 43.58 (0.14) | n.d. | 0.30 (0.08) | 56.11 (0.20) | 0 |
| SF-19 | Si-gl | 5 | 99.46 (0.12) | 0.04 (0.02) | 0.11 (0.01) | 0.37 (0.11) | 0.01 (0.01) |
| SF-19 | fo[a] | 5 | 43.71 (0.09) | n.d. | 0.27 (0.08) | 55.99 (0.09) | 0.01 (0.01) |
| SF-20-A | crs | 5 | 99.81 (0.12) | 0.08 (0.06) | 0.07 (0.02) | 0.01 (0.02) | 0.01 (0.02) |
| SF-20-A | fo | 4 | 41.71 (0.20) | n.d. | 0.01 (0.02) | 58.26 (0.19) | 0.01 (0.01) |
| SF-20-B | Si-gl | 5 | 97.52 (0.17) | 0.35 (0.05) | 0.94 (0.03) | 1.02 (0.08) | 0.14 (0.01) |
| SF-20-B | fo | 4 | 41.61 (0.18) | n.d. | 0.01 (0.01) | 58.35 (0.19) | 0.01 (0.01) |
| SF-21 | fo[a] | 5 | 43.87 (0.04) | n.d. | 0.26 (0.03) | 55.84 (0.04) | 0.01 (0.01) |
| SF-22 | crs | 3 | 99.70 (0.03) | 0.11 (0.01) | 0.13 (0.04) | 0.02 (0.02) | 0.03 (0.03) |
| SF-22 | fo[a] | 5 | 43.43 (0.11) | n.d. | 0.25 (0.01) | 56.28 (0.11) | 0.01 (0.01) |
| SF-23 | fo | 2 | 41.45 (0.09) | n.d. | 0.02 (0.02) | 58.51 (0.10) | 0.01 (0.01) |
| SF-23 | lpx | 3 | 58.67 (0.04) | 0.06 (0.03) | 0.12 (0.09) | 41.11 (0.15) | 0.02 (0.01) |
| SF-24 | fo | 4 | 42.75 (0.13) | n.d. | 0.01 (0.01) | 57.19 (0.16) | 0.03 (0.02) |
| SF-24 | lpx | 5 | 59.85 (0.15) | 0.01 (0.02) | 0.04 (0.03) | 40.05 (0.12) | 0.02 (0.01) |
| SF-27 | fo | 7 | 42.48 (0.21) | n.d. | 0.01 (0.02) | 57.48 (0.22) | 0.01 (0.01) |

| | | | | | | | |
|---|---|---|---|---|---|---|---|
| SF-28 | Si-gl[a] | 8 | 97.98 (0.16) | 0.20 (0.03) | 1.02 (0.08) | 0.74 (0.07) | 0.04 (0.02) |
| SF-28 | fo[a] | 5 | 43.77 (0.06) | n.d. | 0.48 (0.11) | 55.70 (0.07) | 0.02 (0.01) |

Abbreviations: *n*, number of analyses; n.d., not determined.

[a] EDS analyses.
